# Current-Visit and Next-Visit Prediction for Fatty Liver Disease With a Large-Scale Dataset: Model Development and Performance Comparison

**DOI:** 10.2196/26398

**Published:** 2021-08-12

**Authors:** Cheng-Tse Wu, Ta-Wei Chu, Jyh-Shing Roger Jang

**Affiliations:** 1 Department of Computer Science & Information Engineering National Taiwan University Taipei Taiwan; 2 Department of Obstetrics and Gynecology Tri-Service General Hospital, National Defense Medical Center Taipei Taiwan; 3 MJ Health Screening Center Taipei Taiwan

**Keywords:** machine learning, sequence forward selection, one-pass ranking, fatty liver diseases, alcohol fatty liver disease, nonalcoholic fatty liver disease, long short-term memory, current-visit prediction, next-visit prediction

## Abstract

**Background:**

Fatty liver disease (FLD) arises from the accumulation of fat in the liver and may cause liver inflammation, which, if not well controlled, may develop into liver fibrosis, cirrhosis, or even hepatocellular carcinoma.

**Objective:**

We describe the construction of machine-learning models for current-visit prediction (CVP), which can help physicians obtain more information for accurate diagnosis, and next-visit prediction (NVP), which can help physicians provide potential high-risk patients with advice to effectively prevent FLD.

**Methods:**

The large-scale and high-dimensional dataset used in this study comes from Taipei MJ Health Research Foundation in Taiwan. We used one-pass ranking and sequential forward selection (SFS) for feature selection in FLD prediction. For CVP, we explored multiple models, including k-nearest-neighbor classifier (KNNC), Adaboost, support vector machine (SVM), logistic regression (LR), random forest (RF), Gaussian naïve Bayes (GNB), decision trees C4.5 (C4.5), and classification and regression trees (CART). For NVP, we used long short-term memory (LSTM) and several of its variants as sequence classifiers that use various input sets for prediction. Model performance was evaluated based on two criteria: the accuracy of the test set and the intersection over union/coverage between the features selected by one-pass ranking/SFS and by domain experts. The accuracy, precision, recall, F-measure, and area under the receiver operating characteristic curve were calculated for both CVP and NVP for males and females, respectively.

**Results:**

After data cleaning, the dataset included 34,856 and 31,394 unique visits respectively for males and females for the period 2009-2016. The test accuracy of CVP using KNNC, Adaboost, SVM, LR, RF, GNB, C4.5, and CART was respectively 84.28%, 83.84%, 82.22%, 82.21%, 76.03%, 75.78%, and 75.53%. The test accuracy of NVP using LSTM, bidirectional LSTM (biLSTM), Stack-LSTM, Stack-biLSTM, and Attention-LSTM was respectively 76.54%, 76.66%, 77.23%, 76.84%, and 77.31% for fixed-interval features, and was 79.29%, 79.12%, 79.32%, 79.29%, and 78.36%, respectively, for variable-interval features.

**Conclusions:**

This study explored a large-scale FLD dataset with high dimensionality. We developed FLD prediction models for CVP and NVP. We also implemented efficient feature selection schemes for current- and next-visit prediction to compare the automatically selected features with expert-selected features. In particular, NVP emerged as more valuable from the viewpoint of preventive medicine. For NVP, we propose use of feature set 2 (with variable intervals), which is more compact and flexible. We have also tested several variants of LSTM in combination with two feature sets to identify the best match for male and female FLD prediction. More specifically, the best model for males was Stack-LSTM using feature set 2 (with 79.32% accuracy), whereas the best model for females was LSTM using feature set 1 (with 81.90% accuracy).

## Introduction

### Background

Prior research on the use of machine learning for early disease prediction has focused on diabetes, fatty liver disease (FLD), hypotension, and other metabolic syndromes [1]. This study focused on the prediction of FLD, which is widespread in Taiwan, and could lead to liver cirrhosis, fibrosis, and liver cell death. If left untreated for up to 3 years, FLD has a 25% chance of developing into nonalcoholic steatohepatitis and a 10%-15% chance of developing into liver cirrhosis [2,3]. Moreover, FLD increases the prevalence of diabetes, metabolic syndrome, and obesity, creating enormous medical and economic burdens for society. This situation raises an urgent need for early and precise prediction, followed by personalized treatment and lifestyle management. Typically, FLD has been classified into two types according to its cause: alcohol-related fatty liver disease (AFLD) and nonalcoholic fatty liver disease (NAFLD). AFLD is commonly caused by excessive alcohol consumption, whereas NAFLD is due to other more complex factors. Although most prior research has focused on NAFLD prediction rather than AFLD prediction [4-8], there is no inherent reason to conduct separate prediction processes. The previous focus on NAFLD is partly due to the datasets used being insufficiently large to predict both types of FLD. Previous studies have relied on leave-one-out (LOO) cross-validation to avoid overfitting [4-10] on these small datasets. Some prior studies have performed feature selection through human intervention rather than automatic selection [7,11-14], although this is not a common practice in machine learning.

Recently, machine learning has been used extensively in medicine and health care. Dealing with large datasets with many features requires efficient methods to reduce the computing time. We adopted one-pass ranking (OPR) for automatic feature selection, with accuracy similar to the features selected by sequential forward selection (SFS). OPR enables finding good features for current-visit prediction (CVP) and next-visit prediction (NVP). The contributions of this paper can be summarized as follows. First, we compared the performance of OPR and SFS for automatic feature selection, demonstrating that OPR offers great efficiency with decent accuracy when dealing with a large-dimensional dataset. Second, in addition to CVP, we propose the task of NVP, which is much more important for practicing preventive medicine. To our knowledge, this is the first attempt to perform NVP on FLD. Third, we modeled NVP as a sequence classification problem and proposed two feature sets with fixed or variable intervals for the long short-term memory (LSTM) classifier and some of its variants. Before describing the study, we first provide a review of some important prior work on FLD prediction along with a brief overview of automatic feature selection in machine learning.

### Related Work

#### Literature Survey

Table 1 summarizes the differences between this study and prior research. The dataset used in this study is much larger and covers a much longer period. All of the prior research [4-8,11] summarized in Table 1 used smaller datasets, with sample sizes ranging from less than 100 to 11,000 individuals, covering periods ranging from less than 1 year to 2 years at most. Furthermore, most of these studies only used male data for analysis, such as Jamali et al [5], Yip et al [8], and Wu et al [7], with data sizes below 600 individuals. Although Birjandi et al [4], Islam et al [11], and Ma et al [6] used both male and female data for analysis, their data sizes were at most 11,000 individuals, which is still much smaller than the dataset used in this study. The dataset used in this study is far larger than other datasets reported in the literature, and is thus suitable for separate construction of male and female models, which are much more robust and reliable.

**Table 1:** Comparison of prior research and this study for fatty liver disease (FLD) prediction.

Reference	Sample size	Years of study	Feature selection	FLD type	Gender	Next-visit prediction	Data source
Birjandi et al [4]	<1700	2012	Yes	NAFLD^a^	Male/Female	No	Health screening centers
Jamali et al [5]	<100	2012-2014	No	NAFLD	Male	No	Hospital
Yip et al [8]	<1000	2015	Yes	NAFLD	Male	No	Hospital
Islam et al [11]	<1000	2012-2013	Yes	NAFLD/AFLD^b^	Male/Female	No	Hospital
Ma et al [6]	<11,000	2010	Yes	NAFLD	Male/Female	No	Hospital
Wu et al [7]	<600	2009	No	NAFLD/AFLD	Male	No	Hospital
This study	>150,000	2009-2016	Yes	NAFLD/AFLD	Male/Female	Yes	Health screening dataset

^a^NAFLD: nonalcoholic fatty liver disease.

^b^AFLD: alcoholic fatty liver disease.

In various application domains, LSTM has proven to be the state-of-the-art sequence classifier that can achieve better results than classical methods. For instance, Kim et al [15] developed an epidemic disease spread and economic situation model based on LSTM to predict the economic impact of future COVID-19 spread. Pal et al [16] proposed an LSTM framework to predict a country-based COVID-19 risk category at a given time with a dataset from 180 countries. Zhang et al [17] used LSTM to reproduce soil stress-strain behavior, demonstrating better accuracy than other models. For stock price prediction, Sunny et al [18] proposed an LSTM-based framework to forecast stock trends with high accuracy. In surface-guided radiation therapy, Wang et al [19] created a framework to predict internal liver motion signals and external respiratory motion signals, finding that LSTM can achieve better results. Moreover, Qiao et al [20] proposed a high-precision LSTM model to monitor mooring line responses by using the vessel motion as input. The superior performance of LSTM in previous studies motivated us to use this approach for NVP in the context of FLD prediction.

#### Automatic Feature Selection

Automatic feature selection is an important step in machine learning, since it can identify a feature subset to construct a better model while requiring less computing time for training and testing. Automatic feature selection methods can be divided into three categories: wrappers, filters, and embedded methods. Wrapper methods use a classifier to score the feature subsets, which produces accurate results but is time-consuming. Filter methods use a proxy measure instead of accuracy to score a feature subset, which is efficient but does not always produce a good model since the proxy measure does not always relate to classification accuracy [21]. Embedded methods perform feature selection as part of the model construction process, which tends to lie between wrappers and filters in terms of accuracy and computational complexity [22,23]. This study used more accurate wrapper methods for feature selection, including OPR and SFS [24].

Not all approaches covered in the literature use the wrapper methods for feature selection. For example, as shown in in Table 1, Wu et al [7] manually selected only 10 predictor variables, including age, gender, systolic blood pressure, diastolic blood pressure, abdominal girth, glucose AC, triglyceride, high-density lipoprotein cholesterol, serum glutamic-oxaloacetic transaminase-aspartate aminotransferase, and serum glutamic-pyruvic transaminase-alanine aminotransferase, and then derived their weights by information gain without further verifying their ranking by classification accuracy.

### Common Classifiers Used in This Study

This study used different conventional classifiers for CVP, including Adaboost [25], support vector machine (SVM) [26], logistic regression (LR) [27], random forest (RF) [28,29], Gaussian naïve Bayes (GNB) [30], decision tree C4.5 [31], and classification and regression trees (CART) [32]. For NVP, since the input is a variable-length sequence, we used LSTM [33], bidirectional LSTM (biLSTM) [34], Stack-LSTM [35], Stack-biLSTM [36], and Attention-LSTM [37].

## Methods

### Study Design and Process

#### Flowchart

**Figure 1:**
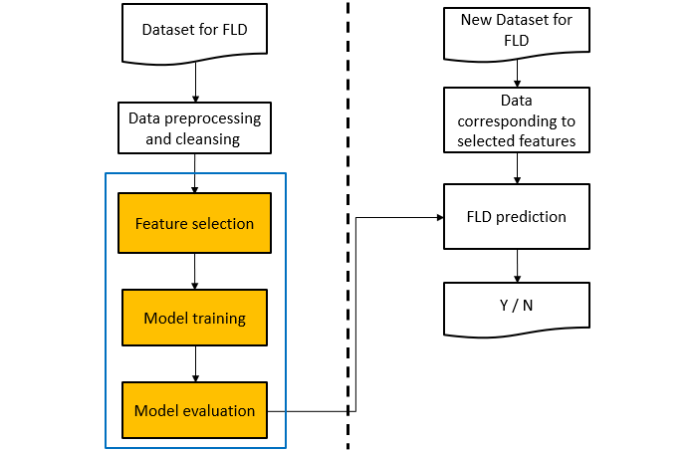
Flowchart of current-visit prediction and next-visit prediction for fatty liver disease (FLD).

**Figure 2:**
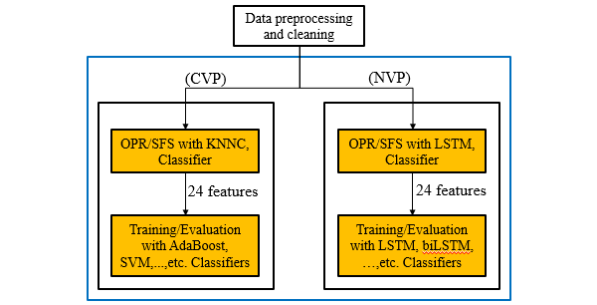
Flowchart of current-visit prediction (CVP) and next-visit prediction (NVP) for fatty liver disease (FLD) with different classifiers. OPR: one-pass ranking; SFS: sequential forward selection; KNNC: k-nearest neighbor classifier; SVM: support vector machine; LSTM: long short-term memory; biLSTM: bidirectional long short-term memory.

This study explored feature selection schemes for CVP and NVP, and proposes two feature sets for NVP using LSTM. Figure 1 shows the flowchart for FLD prediction. First, we needed to perform data preprocessing and cleaning, which is covered in further detail in the Dataset subsection below. We then used different feature selection methods and different classifiers for the two prediction types (CVP and NVP). As shown in Figure 2, we used automatic feature selection (such as OPR or SFS) to select the most critical features from a given classifier, including K-nearest neighbor classification (KNNC), and then adopted a procedure for performance evaluation (such as k-fold cross-validation). Following feature selection, we constructed other more complicated models for prediction and evaluation.

#### CVP Model

Although fatty liver has no special symptoms, there is a certain chance that fatty hepatitis will develop in the long term, and it may progress to serious liver diseases such as cirrhosis, liver failure, and even liver cancer [38,39]. Through the CVP model, the risk of FLD can be predicted directly. For those with a low FLD risk, there is no need to spend time and money in arranging abdominal ultrasound examinations. However, groups with a high risk of FLD are recommended to receive an abdominal ultrasound for early detection and prevention of significant liver diseases. Therefore, CVP can achieve the goal of rapid screening with timely and appropriate intervention, if necessary.

For this task, CVP uses a classifier with all important information (including lab and questionnaire results) at the current visit as inputs to predict whether or not the patient currently has FLD. Correct execution of CVP with selected features can help the doctor better understand what features are more likely to contribute to FLD. Sufficiently high CVP accuracy allows patients with a low FLD risk to forego a time-consuming and costly abdominal ultrasound. That is, CVP can be used for rapid screening at medical clinics that do not have the equipment or specialists needed to manually diagnose FLD. This can effectively reduce staff and equipment requirements at clinics and hospitals, which is of particularly importance in the era of the COVID-19 pandemic.

For CVP feature selection, we used two wrapper-based methods, OPR and SFS, with a simple classifier of KNNC and LOO cross-validation for performance evaluation. Following this rapid feature selection, we used the selected features for model training and evaluation with other advanced classifiers, including Adaboost, SVM, LR, RF, GNB, decision trees C4.5, and CART.

#### NVP Model

Early prediction also plays an essential role in disease prevention, especially for chronic diseases. With NVP, our system can even predict the next visit result, allowing physicians to arrange abdominal ultrasound examinations or other appropriate interventions for patients with a high future risk of FLD. For this task, we used a sequence classifier with all historical information (up to the current visit) as inputs to predict whether or not the patient will be diagnosed with FLD at the next visit. NVP is more important than CVP from the perspective of preventive medicine. If the patient is predicted to have a high probability of FLD risk at the next visit, the physician can suggest lifestyle changes (eg, diet, smoking, alcohol consumption) to effectively modify the key features that contribute to FLD in NVP, along with other appropriate interventions, including abdominal ultrasound at the next health check.

**Figure 3:**
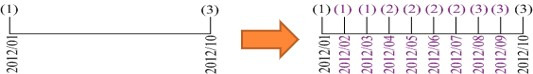
Interpolation for the questionnaire features between any two medical checkups.

For feature selection in NVP, we used OPR with the LSTM classifier and a hold-out test (ie, training and testing) for performance evaluation. Note that we could not use SFS for feature selection since it is too time-consuming for LSTM. If we want to create equal-spaced features for each month between two visits for LSTM, we need to perform linear interpolation between these two visits for each subject. For lab test features (with continuous numerical values), this is achieved by spline interpolation with the piecewise cubic method. For questionnaire features (with categorical values of integers), this is achieved by linear interpolation with rounding off to the nearest labels, as shown in Figure 3.

#### Feature Selection

**Figure 4:**
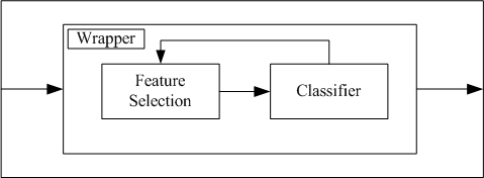
Conceptual diagram of wrappers that interact with a given classifier to select critical features.

As mentioned above, there are three categories of feature selection methods: wrappers, filters, and embedded methods [40]. In general, classification accuracy is strongly dependent on wrapper-selected features; however, this is a time-consuming approach. To strike a balance between efficiency and effectiveness, we compared two wrappers, OPR and SFS, for rapid feature selection based on our large dataset and a given classifier, as shown in Figure 4.

### Dataset

#### General Characteristics of the Dataset

**Figure 5:**
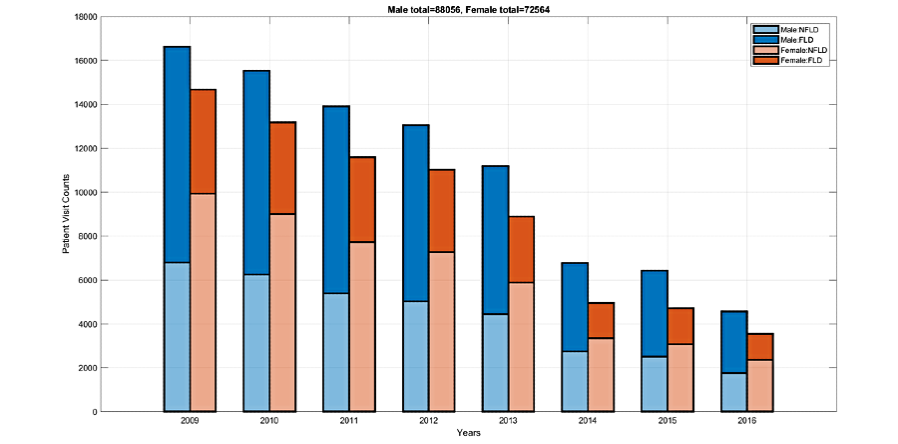
Visit counts for males (blue) and females (red) per year in the MJ-FLD dataset and statistics of no fatty liver disease (NFLD) and fatty liver disease (FLD) per year. The drop from 2013 to 2014 is likely due to the implementation of Taiwan’s Personal Data Protection Act.

This study is primarily related to the MJ-FLD dataset [41], which was collected from a medical checkup clinic in Taipei from 2009 to 2016. This large dataset consists of 160,620 unique (people) visits (88,056 males and 72,546 females) with 446 features (also known as biodata) in total, including 289 from questionnaires and 157 from lab tests. Figure 5 shows the annual visit counts of males and females per year. Our goal is to predict whether a given person has FLD or not at the current and next visits. The following subsections explore the dataset in various ways. The sample sizes indicated refer to the total number of visits for all patients.

#### Data Size Over 8 Years

Figure 5 shows the annual visit counts of males and females per year of the dataset. The large disparity between 2013 and 2014 is likely due to enforcement of Taiwan’s Personal Data Protection Act that set opt-in as the default for participation in medical research.

Therefore, between 2013 and 2014, the male count falls from 11,184 to 6770 (60.53% decrease), and the female count falls from 8896 to 4958 (55.73% decrease). Furthermore, over this 8-year period, the class size ratio of no fatty liver disease (NFLD) vs FLD was 0.66 (34,885 vs 53,171) for males and 2.02 (48,574 vs 23,990) for females. For each year from 2009 to 2016, the class size ratios of NFLD vs FLD were 0.69, 0.67, 0.63, 0.63, 0.66, 0.68, 0.64, and 0.63 for males, and 2.09, 2.16, 2.0, 1.93, 1.94, 2.1, 1.89, and 1.96 for females, respectively (Figure 5). These statistics indicate that the overall dataset is not highly imbalanced, and the class size ratios broken down by gender and year do not vary excessively.

#### Dataset Properties

**Figure 6:**
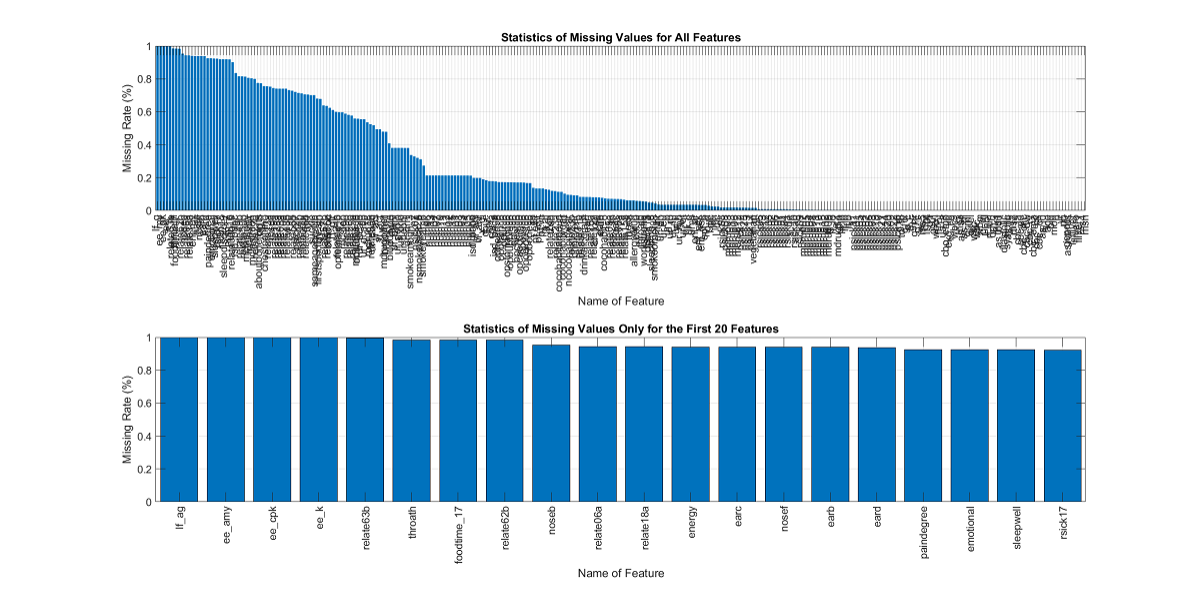
The ratio of missing values for all features and for the top 20 features in the MJ-FLD dataset.

**Figure 7:**
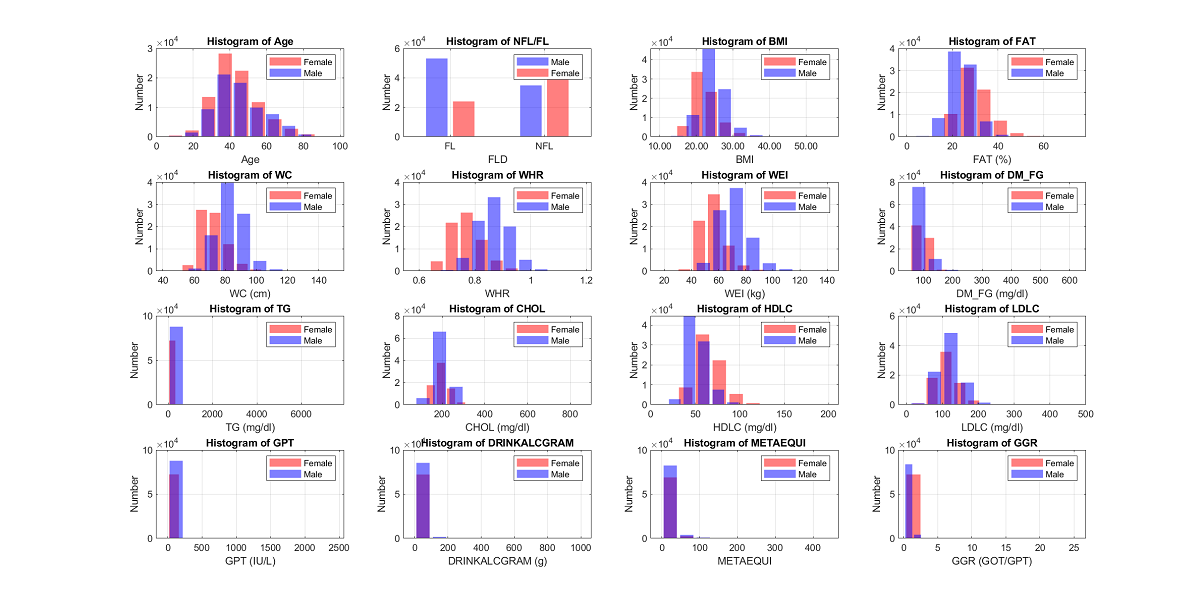
Histograms of important features of the MJ-FLD dataset for males (blue) and females (red). NFL: no fatty liver; FL: fatty liver; FAT: body fat; WC: waist circumference; WHR: waist-to-hip ratio; WEI: weight; DM_FG: diabetes for fasting glucose; TG: triglyceride; CHOL: total cholesterol; HDLC: high-density lipoprotein cholesterol; LDLC: low-density lipoprotein cholesterol; GPT: serum glutamic-pyruvic transaminase; DRINKALCGRAM: alcohol per gram; METAEQUI: metabolic equivalent for exercise per week; GGR: serum glutamic-oxaloacetic transaminase to glutamic-pyruvic transaminase ratio.

Another characteristic of the dataset is its high ratio of missing values, as shown in Figure 6, which plots the percentage of missing values for all features and the top 20 features. Since the features with missing value ratios of 90% or higher are hard to impute, these 17 features were eliminated, leaving 252 features for further processing. The histograms of important features for males and females are shown in Figure 7. Some features such as waist-hip ratio displayed very different gender-dependent histograms.

#### BMI Progression Over 8 Years

**Figure 8:**
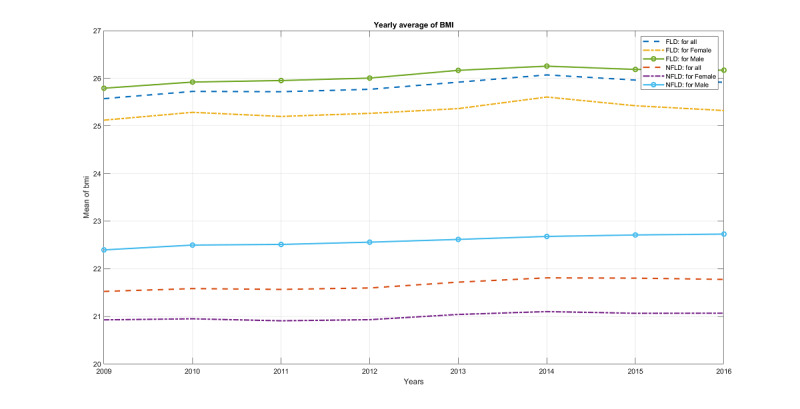
Progression of yearly average BMI over 8 years, broken down by [FLD, NFLD] x [male, female, overall] into 6 curves. FLD: fatty liver disease; NFLD: no fatty liver disease.

Some features such as BMI are strong indicators of FLD. Figure 8 plots the yearly average BMI for FLD and NFLD, broken down by males, females, and overall. Six curves are clearly divided into two groups of FLD and NFLD, with BMI for FLD consistently higher than that of NFLD. Within the same class (FLD or NFLD), males usually have a higher BMI than females. Moreover, the three curves for FLD show higher variance than the other three curves for NFLD, indicating that FLD patients might have a more dramatic BMI progression.

#### Data Preprocessing

Our dataset is based on health screening results from individuals, some of whom underwent multiple screenings at different intervals with different sets of screening items. As a result, there are several missing values in the dataset that needed to be imputed before further processing. Moreover, the questionnaires also changed over these 8 years when the dataset was compiled; therefore, we needed to consolidate the answers to different questionnaires of the same type.

To perform missing values imputation in our dataset, we used the mean for numerical features and the mode for questionnaire features. This is a quick and dirty method, especially for such a large dataset. Missing value imputation could be accomplished using other more complicated methods such as MICE (Multivariate Imputation by Chained Equations) [42], which imputes each missing value sequentially by another machine learning method. The process iterates until all of the imputed values converge, which usually takes a long time and is thus not feasible for a large dataset with many missing values.

To consolidate the answers to different questionnaires of the same type in the dataset, we needed to use some heuristics to derive consistent numerical values as features for machine learning. For instance, “grams of alcohol” represents the average weekly alcohol intake in grams [43,44], which was derived by combining some questionnaire items related to drinking from the MJ-FLD dataset. Similarly, to derive “weekly exercise metabolic equivalent,” we needed to combine some questionnaire items related to exercise.

In summary, the steps involved in data preprocessing were performed as follows:

1. Deletion of useless features: Our first step in data preprocessing was to drop features that are apparently not related to FLD, such as “cervical cancer,” “prostate cancer,” “other forms of cancer,” “other hereditary diseases,” “Chinese medicine,” and “has your mother or sister had breast cancer, ovarian cancer, or endometrial cancer?”
2. Missing value handling: Missing values in the dataset were replaced by the average for numerical features and by the mode for categorical features.
3. Feature conversion: To create consistent features from questionnaires, we consolidated highly related questionnaires and expressed the corresponding responses in numeric terms. For example, the feature “grams of alcohol consumption” was derived from responses to the questionnaire items “type of drink,” “amount of drink,” “drink or not,” and “alcohol density.” Similarly, the feature “weekly exercise metabolic equivalent” was derived from responses to the questionnaire items “type of sport,” “frequency of sport,” and “time for sport.”
4. Deletion of redundant features: Some highly redundant features were deleted from the dataset, such as “BMI,” “systolic/diastolic blood pressure while lying down left arm,” and “systolic/diastolic blood pressure while lying down right arm.”
5. Feature-wise normalization: This was achieved by z-score normalization to have a zero mean and unit variance for each feature:

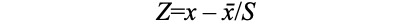



where 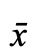 is the sample mean of feature *x* and *S* is the sample standard deviation of feature *x*.

### Environment and Specification

All experiments were performed on a 64-bit Windows-10 server, with an Intel Xeon Silver 4116 CPU at 2.10 GHz, two NVIDIA Quadro GV100 GPUs, 256 GB RAM, 1-TB hard disk, and Matlab R2020b (9.8.0.1538559), and python 3.8.2, scikit-learn 0.24.1, TensorFlow-GPU 2.4.1.

All of the models in this study were constructed based on the MJ-FLD dataset [41]. Each of our experiments was designed with the goal of finding something meaningful in the dataset; therefore, we may use different ways to partition the dataset into subsets for training, validation, and testing for different experiments. We also performed necessary dataset preprocessing before using the data for modeling, including missing value imputation, feature consolidation, and feature-wise *Z*-score normalization, as explained above.

## Results

### Feature Selection With Various Methods

To investigate the effectiveness of different feature selection methods, we compared the computer-selected features with expert-suggested FLD features. All of the expert-suggested features are listed in Table 2, with a brief explanation for each. For instance, the well-known high-risk factors (or features) suggested by domain experts included BMI, body fat, and waist circumference. The critical factors related to AFLD are also listed, including “drinkalcgram” (average alcohol consumption in grams) and “drinkyear” (how many years the patient has been drinking alcohol).

**Table 2:** Features of fatty liver disease, including those suggested by domain experts or selected by one-pass ranking (OPR) and sequential forward selection (SFS) for current-visit prediction and next-visit prediction.

Features	Explanation	Suggested by experts	OPR			SFS			OPR (Feature set 1)			OPR (Feature set 2)	
			Selected by OPR	Match^a^	Selected by SFS		Match	Selected by OPR		Match	Selected by OPR		Match
age	Age				✓			✓			✓		
blood type	Blood type				✓								
bmd	Bone mineral density				✓			✓			✓		
bmi	Body mass index	✓	✓	✓	✓		✓	✓		✓	✓		✓
cc (cm)	Chest circumference		✓					✓					
cci (cm)	Chest circumference during inspiration		✓					✓			✓		
cea (ng/ml)	Carcinoembryonic antigen				✓								
ch	The ratio of chol/hdlc	✓	✓	✓				✓		✓	✓		✓
chol (mg/dl)	Total cholesterol	✓			✓		✓						
diastolic	Diastolic blood pressure							✓					
drinkalcgram (g)	Alcohol per gram	✓											
drinkyear	How many years have you been drinking?	✓											
e (%)	Eosinophils										✓		
ery (10^6^/µl)	Red blood cells				✓								
fat (g)	Body fat	✓	✓	✓	✓		✓	✓		✓	✓		✓
fg (mg/dl)	Diabetes mellitus fasting glucose	✓	✓	✓	✓		✓	✓		✓	✓		✓
food18	How many servings of bread do you eat?	✓											
food19	Do you add jam or honey to your food?	✓											
food20	Do you add sugar to your coffee, tea, cola/soda, fruit juices, or other beverages?	✓											
food21	How many servings of your food intake are fried in oil?	✓			✓		✓						
ggr	The ratio of got/gpt	✓	✓	✓	✓		✓	✓		✓	✓		✓
ggt (IU/L)	Gamma-glutamyl transferase		✓		✓			✓			✓		
got (IU/L)	Serum glutamic-oxaloacetic transaminase (sGOT)		✓		✓			✓			✓		
gpt (IU/L)	Serum glutamic-pyruvic transaminase (sGPT)	✓	✓	✓				✓		✓	✓		✓
hc (cm)	Hip circumference		✓		✓			✓			✓		
hdlc (mg/dl)	High-density lipoprotein cholesterol	✓	✓	✓	✓		✓	✓		✓	✓		✓
hei (cm)	Height				✓						✓		
hema (%)	Hematocrit										✓		
Ldlc (mg/dl)	Low-density lipoprotein cholesterol	✓						✓		✓			
leu (10^3^/ml)	White blood cells							✓			✓		
mcv (fl)	Mean corpuscular volume				✓								
mdrug10	Steroids	✓			✓		✓						
mdrug8	Medicine for asthma	✓			✓		✓						
metaequi	Metabolic equivalent for exercise per week	✓									✓		✓
n (%)	Neutrophils										✓		
p (mg/dl)	Phosphorus		✓										
pul (beat/mint)	Pulse rate							✓			✓		
relate33b	In the last 3 months, have you lost weight by more than 4 kg?	✓											
relate17a	Have your defecation habits changed?		✓										
sdephi (/HPF)	Sediment epithelial cells high		✓										
sdrhi (/HPF)	Sediment red blood cells high		✓										
sdwhi (/HPF)	Sediment white blood cells high		✓										
sg	Specific gravity		✓										
smokeornot	Have you ever smoked?	✓											
systolic	Systolic blood pressure							✓					
tb (mg/dl)	Total bilirubin										✓		
tg (mg/dl)	Triglyceride	✓	✓	✓	✓		✓	✓		✓	✓		✓
tp (g/dl)	Total protein							✓					
tsh (µIU/ml)	Thyroid stimulating hormone		✓		✓								
ua (mg/dl)	Uric acid							✓			✓		
vanl	Visual acuity (naked left eye)				✓								
wc (cm)	Waist circumference	✓	✓	✓	✓		✓	✓		✓	✓		✓
wei (kg)	Weight	✓	✓	✓	✓		✓	✓		✓			
Whr	Waist-to-hip ratio	✓	✓	✓				✓		✓			
Workstreng	What is your level of activity at work?		✓										

^a^Indicates a match with the features selected by domain experts based on the literature.

### Intersection Over Union and Coverage

To evaluate the similarity between the feature sets manually selected by human experts (set S1) and automatically selected by OPR/SFS (set S2), we used two similarity indices, intersection over union (IoU) and coverage, defined as follows:

IoU(S1, S2)=|S1∩S2|/|S1∪S2|

Coverage(S1, S2)=|S1∩S2|/|S1|

Both similarity indices range from 0 to 1, and a higher value indicates higher similarity.

### Experiment 1: CVP With Optimum Years of Training Data and Feature Selection

**Figure 9:**
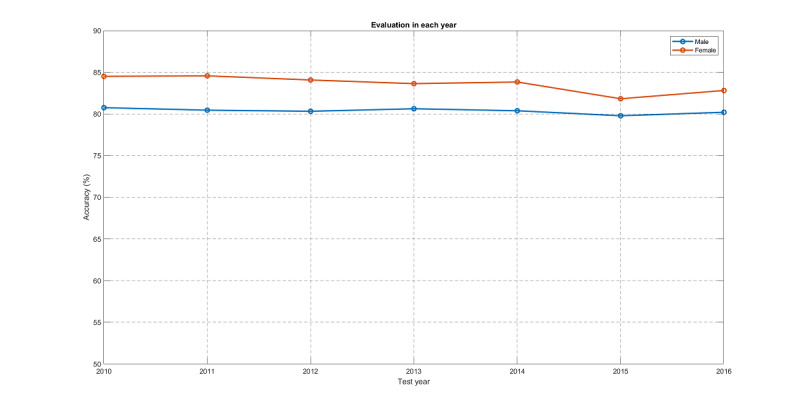
Test accuracy for each year using the previous year data for training and the current year data for testing for both males and females.

Given the size of the dataset, we can explore it in different directions. First, we needed to confirm the modeling accuracy of CVP across years, which was achieved using the previous year data for training and the current year data for testing. The test accuracy for each year is shown in Figure 9.

**Figure 10:**
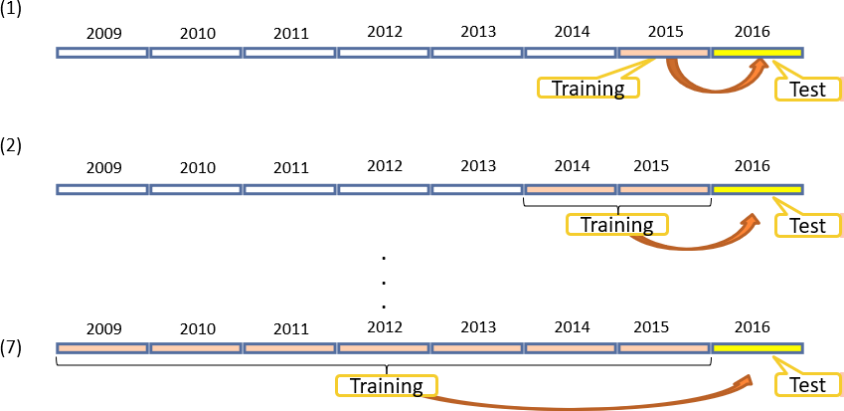
The models of seven subtasks for training in intervals (2015, 2014-2015, 2013-2015, 2012-2015, 2011-2015, 2010-2015, 2009-2015) and 2016 for testing, for males.

Next, we wanted to further explore the optimum duration in years considered for modeling in feature selection. In general, using a long period of historical data for modeling may result in mismatching with the test data since the optimum model may change over time. However, a short period of historical data may not be sufficient for stable model construction. As a result, we needed to identify the optimum duration in years where the training data are obtained for predicting the data in 2016. More specifically, we defined seven subtasks for training data in intervals (2015, 2014-2015, 2013-2015, 2012-2015, 2011-2015, 2010-2015, 2009-2015), and the test data were from 2016. This arrangement is illustrated in Figure 10. Moreover, we performed feature selection for each subtask to select the best features. The modeling specifications are as follows: dataset, male part of the MJ-FLD dataset; classifier, KNNC; feature selection, OPR with LOO cross-validation for the performance index to select the most important 24 features (this number was used to match the number of features suggested by the domain experts.)

**Figure 11:**
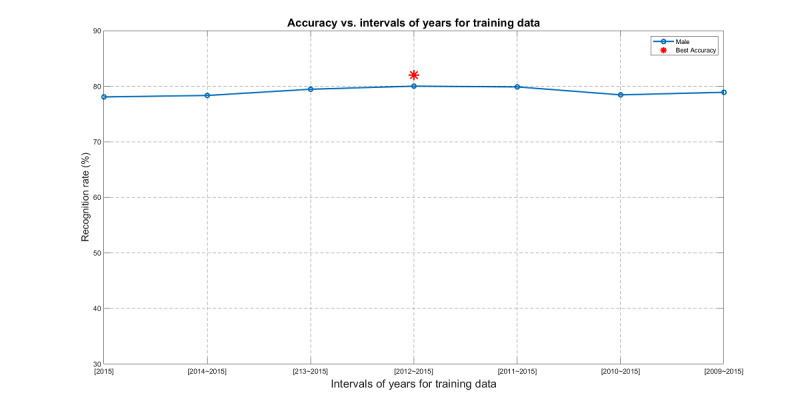
The best year interval for the model of male fatty liver disease prediction is 2012-2015.

**Figure 12:**
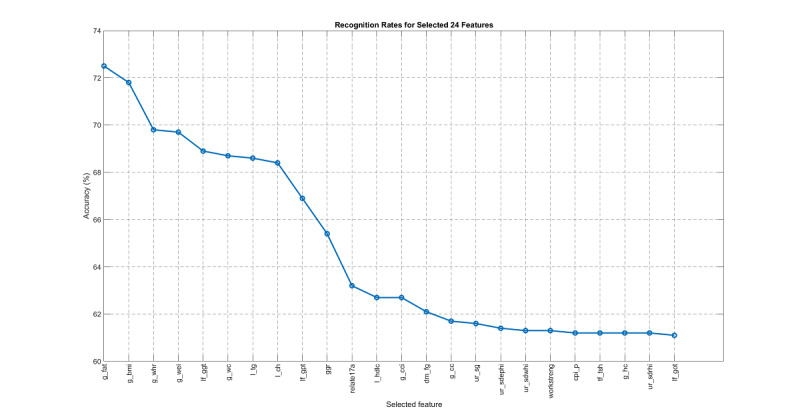
Features selected by one-pass ranking based on the standard set, in descending order of recognition rate.

The result is shown in Figure 11, where the best interval was 2012-2015, achieving the best test accuracy of 80.00%. The corresponding OPR-selected features are shown in Figure 12. For comparison, if we used the same training/test pair to evaluate SFS-selected and expert-suggested 24 features, the accuracies were 78.37% and 79.78%, respectively. Using the same evaluation steps on female data produced the same result; that is, the best interval was 2012-2015.

For easy reference, we refer to the training set of the interval 2012-2015 and the test set from 2016 as the “standard set.” Based on the standard set, we applied OPR and SFS, as shown in Table 3. The result indicated that SFS is slightly better than OPR in terms of classification accuracy (80.92% vs 80.32%). In terms of the selected features, SFS was also slightly better than OPR, with 50.00% vs 45.83% for coverage rate and 33.33% vs 29.73% for IoU. However, SFS achieved these marginal improvements at the cost of computing time, which was approximately three times slower than that of OPR. The features selected by OPR, SFS, and domain experts are listed in Table 2, including the most common features for FLD with a simple explanation. In the table, any matched features selected by OPR or SFS are indicated with a check mark in the “Match” column.

**Figure 13:**
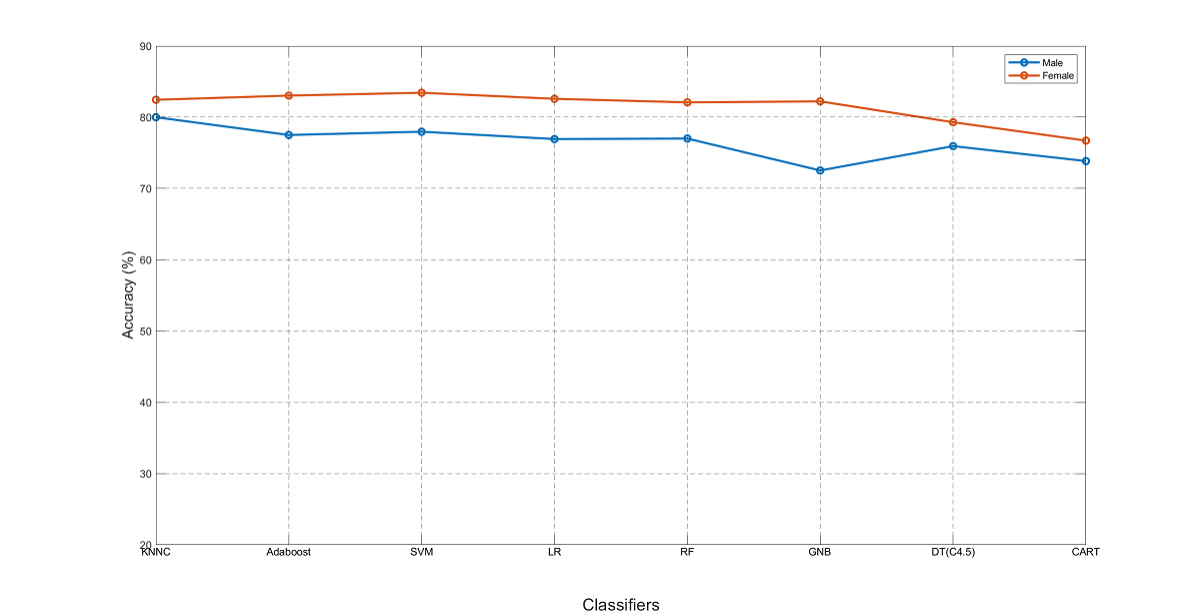
Performance of various classifiers on the standard set. KNNC: k-nearest neighbor classifier; SVM: support vector machine; LR: logistic regression; RF: random forest; GNB: Gaussian naive Bayes; CART: classification and regression trees.

Finally, we tested other classifiers on the standard set, including KNNC, Adaboost, SVM, LR, RF, GNB, decision trees C4.5, and CART, as shown in Figure 13. The classifiers of Adaboost and SVM showed higher accuracy than the others. We also noticed that for all classifiers, the accuracy for the females outperformed that for the males, which will be discussed in the next subsection. The area under the receiver operating characteristic curve (AUROC), precision, recall, and F1 scores for these 7 classifiers are shown in Table 4. In particular, the AUROC values for these classifiers for CVP were all higher for females than for males.

**Table 3:** Comparison of one-pass ranking (OPR) and sequential forward selection (SFS) in terms of feature selection and classification.

Metric		OPR		SFS
**Feature selection**				
	Intersection over union	29.73% (11/37)	33.33% (12/36)	
	Coverage	45.83% (11/24)	50.00% (12/24)	
Classification accuracy		80.32%		80.92%

**Table 4:** Performance metrics for eight different classifiers.

Classifier		AUROC^a^	Precision	Recall	F1 score	Accuracy
**KNNC^b^**						
	Males	0.80	0.77	0.82	0.79	80.00%
	Females	0.87	0.77	0.68	0.72	82.45%
**Adaboost**						
	Males	0.85	0.80	0.85	0.82	77.51%
	Females	0.90	0.77	0.70	0.74	83.07%
**SVM^c^**						
	Males	0.85	0.80	0.86	0.83	77.97%
	Females	0.90	0.80	0.69	0.74	83.44%
**LR^d^**						
	Males	0.85	0.83	0.78	0.81	76.94%
	Females	0.90	0.71	0.82	0.76	82.59%
**RF^e^**						
	Males	0.85	0.83	0.79	0.81	77.01%
	Females	0.90	0.72	0.80	0.76	82.90%
**GNB^f^**						
	Males	0.79	0.83	0.70	0.76	72.53%
	Females	0.88	0.77	0.67	0.72	82.23%
**DT^g^ (C4.5)**						
	Males	0.83	0.83	0.76	0.80	75.95%
	Females	0.87	0.67	0.78	0.72	79.30%
**CART^h^**						
	Males	0.73	0.79	0.78	0.79	73.85%
	Females	0.76	0.63	0.75	0.68	76.72%

^a^AUROC: area under the receiver operating characteristic curve.

^b^KNNC: k-nearest-neighbor classifier.

^c^SVM: support vector machine.

^d^LR: logistic regression.

^e^RF: random forest.

^f^GNB: Gaussian naïve Bayes.

^g^DT: decision tree.

^h^CART: classification and regression trees.

### Experiment 2: Hormonal Influence in CVP

**Figure 14:**
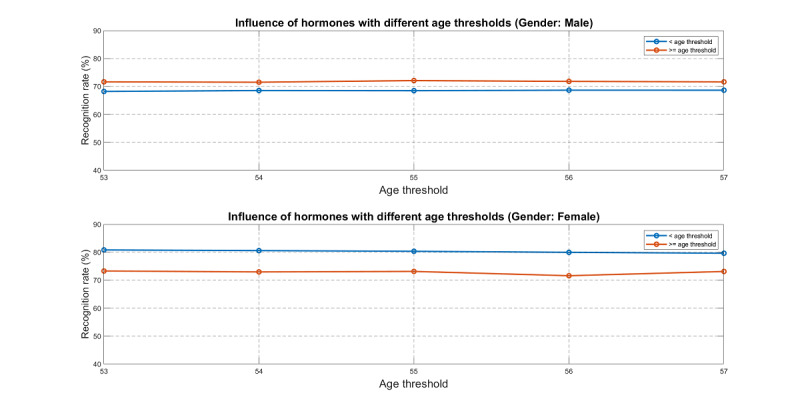
Investigation of hormonal influence, assuming menopause/andropause occurs at ages 53, 54, 55, 56, and 57, respectively. The upper plot is for males and the lower plot is for females. Each yellow-purple bar pair indicates the accuracy before and after menopause at a specific age. The dataset used for this analysis corresponds to the years 2009-2016.

As shown in Figure 13, the accuracy for females was consistently higher than that for males. This may be due to data imbalance, which is further addressed in the Discussion section. Moreover, we can also explore the influence of hormones for both males and females in CVP. To this end, we assumed that menopause/andropause occurs at a certain age and then performed modeling/evaluation before and after the age to determine the difference in prediction accuracy. More specifically, we split the whole dataset (2009-2016) into two subsets, “before” and “after,” according to the assumed age of menopause. Within each subset, the period of 2009-2015 was used for training and 2016 was used for testing with the naïve Bayes classifier. The results are shown in Figure 14, in which we assumed that menopause/andropause occurs at ages 53, 54, 55, 56, and 57, and derived the accuracy before and after menopause/andropause for both males and females. We observed that the “before” accuracy is consistently higher than that of “after” for females. Moreover, the accuracy differences between “before” and “after” were much higher for females than for males. This is because female hormones can maintain the basal metabolic rate at a certain level before menopause such that the accumulation of fat in the internal organs is less likely to occur, thus improving the FLD prediction accuracy. After menopause, women do not have normal hormone secretion, leading to a less balanced body status and more challenging FLD prediction. For fatty liver, lifestyle intervention is usually recommended for treatment. Chalasani et al [45] reviewed several population-based studies and pointed out that because body fat, sex hormone metabolism, and lifestyle have gender differences, the occurrence of FLD will vary by gender [46]. Therefore, we believe that the accuracy of CVP will also differ due to these indicators.

Figure 14 shows that the difference in recognition rate for males does not change obviously between the “before” and “after” age threshold, but it does for females within each subset. This means that sex hormones play an important role in FLD prediction for females. In other words, the greater the effect of sex hormones will result in a higher recognition rate for prediction.

For females, sex hormones will be affected not only by the lifestyle habits an individual engages in to maintain a good figure but also by factors such as dieting and drugs. To achieve a slim figure, many women try various types of diets that have several side effects, which may affect specific biochemical tests related to FLD. In addition, some women may resort to the ingestion of nutritional supplements or other forms of “diet pills” to lose weight. However, many of these drugs contain unknown ingredients or illegal substances that could significantly affect the results of tests associated with FLD.

### Experiment 3: LSTM for NVP

In this experiment, we used LSTM with various setups for NVP. LSTM is a well-known sequence classifier that can use information from historical visits, with no length limit, to predict the possibility of FLD at the patient’s next clinic visit. As explained earlier, from the perspective of preventive medicine, NVP is much more important than CVP. The specifications for feature selection of NVP are as follows: dataset, male subjects in the MJ-FLD dataset; classifier, LSTM; feature selection, OPR with 3-fold cross-validation to select the most important 24 features.

**Figure 15:**
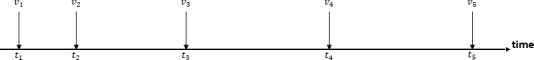
A typical visit pattern and the extracted input/output pairs for training long short-term memory (LSTM). If the visit pattern is denoted by [v1, v2, v3, v4, v5], then we can extract 4 input/output pairs for training LSTM: {v1⇒v2}, {v1,v2⇒v3}, {v1, v2, v3⇒v4}, {v1,v2,v3,v4⇒v5}. Note that patients with only a single visit are discarded in this next-visit prediction task.

**Figure 16:**
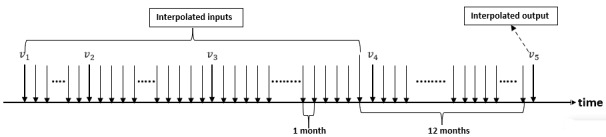
To create fixed-interval data for feature set 1, we need to perform interpolation on the input/output parts. For this case, the input part is interpolated to have a fixed interval of 1 month and the output part is interpolated to have a time distance of 12 months from the nearest time of the input.

**Figure 17:**
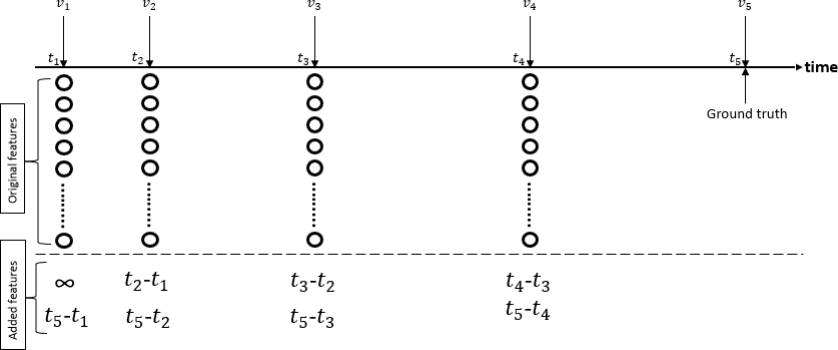
To create variable-interval data for feature set 2, we need to add two extra inputs to long short-term memory, including the time span from the previous visit and the time span to the future point at which the prediction occurs.

In general, clinic visits do not always occur at regular intervals. For a given visit pattern of length N, we can extract N – 1 input-output pairs for NVP modeling using LSTM, as shown in Figure 15 where N=5. To deal with this situation of nonregular intervals, we designed two types of LSTM that have two types of feature sets. In feature set 1 with fixed intervals, interpolation was performed to obtain a fixed-interval input sequence to our sequence classifier. For instance, the input can have a fixed interval of 1 month and the output can be 12 months into the future, as shown in Figure 16. If the next visit is less than or equal to 12 months away from the current visit, then we can easily perform interpolation for the input. However, if the next visit is more than 12 months away from the current visit, then we simply duplicate the data at the current visit to the subsequent months until we have enough data to perform NVP. In feature set 2 with variable intervals, we used the visit pattern directly with extra inputs to preserve the interval information and target time for prediction. For instance, if we have *d* features for a visit, then the number of inputs should be *d*+2, with the additional first feature indicating the time span from the previous visit and the additional second feature indicating how far in the future the prediction should be made, as shown in Figure 17.

For feature set 1 with fixed-interval data, the dataset included the number of input/output pairs for males (13,315) and for females (10,998). The mean input sequence length for males and females was 42.03 (SD 21.25, range 5-96) and 41.44 (SD 20.84, range 4-96), respectively. For feature set 2 with variable-interval data, there were 16,081 input/output pairs for males with a mean input sequence length of 3.32 (SD 1.46, range 2-13), and 13,364 input/output pairs for females with a mean input sequence length of 3.15 (SD 1.35, range 2-15).

Feature set 2 with input data from variable intervals showed three major advantages: (1) the unfolded LSTM network has considerably fewer stages, resulting in much shorter training and prediction times; (2) the dataset is used directly with no need to perform extra interpolation in advance, thus reducing time requirements and increasing precision; and (3) it can perform any prediction at any time in the future directly.

First, the input/output pairs used to train feature set 1 (with 24 features for males in the dataset) were prepared as follows. All patients with only a single visit were removed from the dataset, reducing the total number of males from 34,856 to 22,972. From the historical data for each patient, we interpolated data between any two consecutive visits to the monthly values. For a specific visit (excluding the last one), the first 12 months of the interpolated data right before the visit were used as the feature set 1 input, while the interpolated output at 12 months right after the visit was used as the output. The input-output data pairs were then collected using moving windows with a stride of 1 month.

**Figure 18:**
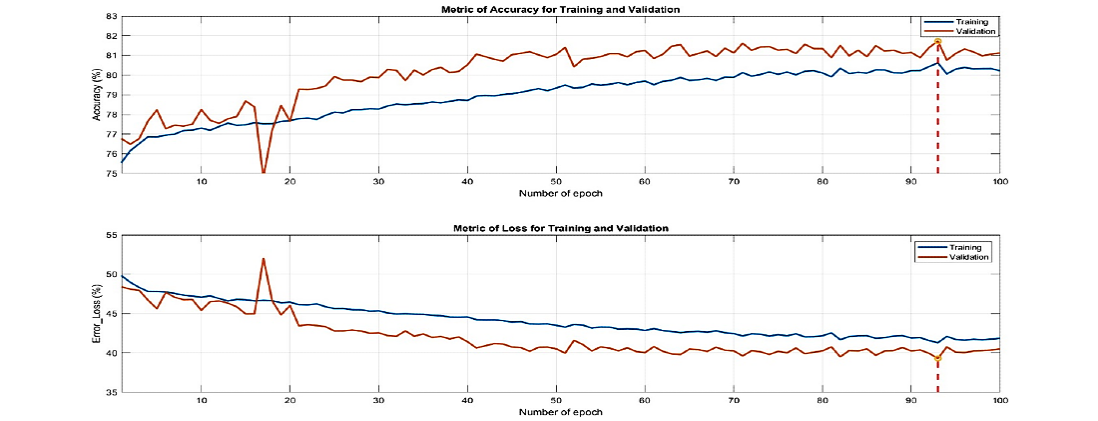
The accuracy (upper plot) and loss (lower plot) for training and validation during the training of feature set 1 for male subjects of the MJ-FLD dataset. The best model was selected at epoch 93 where the validation accuracy reached its maximum of 81.72%.

The final count of input-output data pairs for trained feature set 1 with 24 features was 469,159. These data pairs were divided into 70% used for training (10% of which was used for validation) and 30% used for testing, all with stratified partitioning. All training options and parameters for LSTM are listed in Multimedia Appendix 1. Figure 18 shows the training and validation accuracy/loss vs epochs during the training process. As usual, the best model was selected at the epoch where the validation loss reached its minimum or the validation accuracy reached its maximum. In this case, the best model was selected at epoch 93 where the validation accuracy reached its maximum of 81.72%.

**Figure 19:**
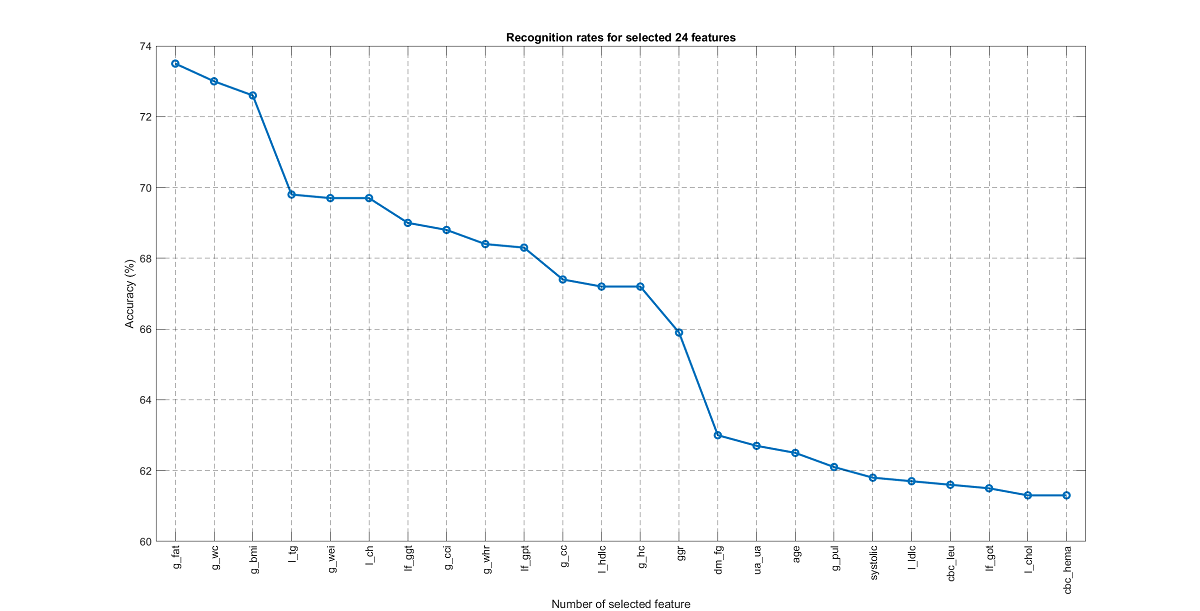
Features selected by one-pass ranking based on feature set 1, ranked by accuracy.

Based on the above process, we then performed OPR on top of feature set 1 to derive 24 features. As shown in Figure 19, when compared with the expert-selected features, the OPR-selected features achieved an IoU of 29.73% and a coverage of 45.83%, which is satisfactory based on the opinions of the domain experts we consulted. By contrast, the OPR on top of feature set 2 achieved an IoU of 23.08% and the coverage was 37.50%. All results for feature sets 1 and 2 are shown in Table 5. The AUROC, precision, recall, and F1 scores are shown in Table 6.

**Table 5:** Comparison of intersection over union (IoU), coverage, and accuracy of the features selected by one-pass ranking (OPR) and domain experts in the two feature sets.

Metric	OPR		Experts	
	Feature set 1	Feature set 2	Feature set 1	Feature set 2
IoU	29.73% (11/37)	23.08% (9/39)	N/A^a^	N/A
Coverage	45.83% (11/24)	37.50% (9/24)	N/A	N/A
Accuracy	75.91%	77.32%	75.40%	74.95%
Computing time (seconds)	5875	1452	N/A	N/A

^a^N/A: not applicable.

**Table 6:** Comparison of performance, computing time, and error reduction rate with five long short-term memory (LSTM)-based classifiers.

Classifier				AUROC^a^		Precision		Recall		F1 score		Accuracy		Computing time (s)		Error reduction rate
**LSTM**																
	**FSI^b^**															
		Males	0.83		0.75		0.74		0.75		76.54%		2713		5.33%	
		Females	0.88		0.80		0.78		0.79		81.90%		2485		30.86%	
	**FS2^c^**															
		Males	0.86		0.78		0.77		0.78		79.29%		1466		16.42%	
		Females	0.87		0.79		0.77		0.78		80.81%		1469		26.70%	
**biLSTM^d^**																
	**FS1**															
		Males	0.83		0.75		0.74		0.75		76.66%		3380		5.81%	
		Females	0.88		0.81		0.78		0.79		81.70%		3155		30.10%	
	**FS2**															
		Males	0.87		0.78		0.77		0.78		79.12%		1789		15.74%	
		Females	0.88		0.79		0.77		0.78		80.79%		1800		26.63%	
**Stack-LSTM**																
	**FS1**															
		Males	0.84		0.76		0.75		0.75		77.23%		3764		8.11%	
		Females	0.88		0.80		0.78		0.79		81.87%		3524		30.75%	
	**FS2**															
		Males	0.87		0.78		0.77		0.78		79.32%		1952		16.55%	
		Females	0.87		0.79		0.77		0.78		80.51%		2016		25.55%	
**Stack-biLSTM**																
	**FS1**															
		Males	0.84		0.76		0.75		0.75		76.84%		6085		6.54%	
		Females	0.88		0.80		0.78		0.79		81.77%		5429		30.37%	
	**FS2**															
		Males	0.87		0.78		0.77		0.78		79.29%		2714		16.42%	
		Females	0.88		0.79		0.77		0.78		80.78%		2802		26.59%	
**Attention-LSTM**																
	**FS1**															
		Males	0.84		0.83		0.80		0.81		77.31%		N/A^e^		8.43%	
		Females	0.89		0.69		0.79		0.74		80.81%		N/A		26.70%	
	**FS2**															
		Males	0.87		0.87		0.77		0.82		78.36%		N/A		12.67%	
		Females	0.89		0.70		0.81		0.75		81.46%		N/A		29.18%	

^a^AUROC: area under the receiver operating characteristic curve.

^b^FS1: feature set 1.

^c^FS2: feature set 2.

^d^biLSTM: bidirectional long short-term memory.

^e^N/A: not applicable.

**Figure 20:**
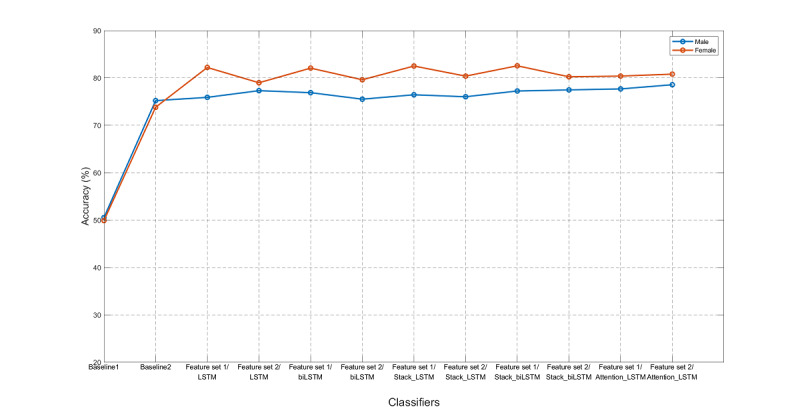
Accuracy for two baseline models and 10 long short-term memory (LSTM) models for males and females. biLSTM: bidirectional LSTM.

We next compared the performances of feature sets 1 and 2 to two baseline models, as shown in Figure 20. The predictor for baseline 1 always outputs the class with a larger percentage in the ground truth. In the case of the MJ-FLD dataset, the output is always NFLD. Baseline 2 is a simple inference model that always outputs the class of the previous visit. In other words, the prediction is based on the ground truth of the previous visit.

The test accuracy of NVP using feature set 1 (with fixed intervals) and feature set 2 (with variable intervals) for males was 77.31% with Attention-LSTM (8.43% error reduction) and 79.32% with Stack-LSTM (16.55% error reduction), respectively. The error reduction rates were compared with a baseline model of simple inference. For females, the corresponding values were 81.90% with LSTM (30.86% error reduction) and 81.46% with Attention-LSTM (29.18% error reduction). The error reduction rates of four classifiers for males and females are listed in Table 6.

Table 5 shows the IoU and coverage rates of OPR-selected features based on feature sets 1 and 2. The accuracy of feature set 2 was comparable with that of feature set 1 for both males and females. However, the training times were 5875 and 1452 seconds, respectively, indicating that the proposed feature set 2 provides much better efficiency. Note that it is almost impossible to perform SFS in this case due to its lengthy computation. Moreover, for both feature sets 1 and 2, the accuracy results of OPR-selected features (78.20% and 76.79%) were higher than those of the expert-selected features (75.40% and 74.95%), indicating the feasibility of OPR for feature selection of a large dataset with a complex model of LSTM.

For feature set 2, we discarded patients with a single visit to obtain 76,172 input-output pairs; therefore, the number of male patient visits dropped from 34,856 to 22,972. The results of OPR-selected features are listed in Table 2 for comparison. Note that the table does not include feature set 2–based SFS, simply because the computational time for SFS with feature set 2 takes more than 7 days.

## Discussion

### Principal Findings

The computing time of OPR was much lower than that of SFS; however, it can achieve comparable performance (in terms of the overlap between the automatically selected features and the manually selected features) as SFS, especially when dealing with a large-scale dataset with high-dimensional features. The best model for CVP was KNNC for males (80.00%) and SVM for females (83.44%). The best model for NVP was Stack-LSTM using feature set 1 (79.32%) for males and LSTM using feature set 2 (81.90%) for females.

For NVP, the proposed feature set 2 is highly flexible and can achieve comparable results to those obtained with feature set 1; however, the computing time is much shorter, and the prediction can be derived at any time in the future. Both feature sets 1 and 2 outperformed a simple inference model (baseline 2), achieving an error reduction of 16.53% (Stack-LSTM) for males and 30.86% (LSTM) for females.

As shown in Table 4, by comparing two rows of SVM/male and KNNC/male, we can observe that SVM outperformed KNNC in all metrics except for accuracy. As a result, for males, SVM can be used to replace KNNC if accuracy is not the only concern. According to Figure 9 and Figure 13, the CVP for females was consistently better than that for males. This is simply due to the fact that the female dataset is more imbalanced than the male dataset. To demonstrate this, we computed the imbalance factors (data size of the bigger class divided by that of the smaller class) across 8 years: (1.45, 1.49, 1.58, 1.60, 1.52, 1.47, 1.57, 1.60) for males and (2.09, 2.16, 2.0, 1.93, 1.94, 2.1, 1.89, 1.96) for females. Therefore, the imbalance factors for females are consistently higher than those for males, leading to better accuracy for the female dataset.

For CVP, the influence of hormones for females was more intense than that for males, leading to difficulty in FLD prediction for females after menopause, as shown in Figure 14, where the difference in accuracy before and after menopause age is more dramatic for females than for males. In other words, hormones play an important role for FLD prediction in females. However, after menopause, women lose protection from sex hormones, which can increase the risk of chronic and/or metabolic diseases. This would make FLD prediction harder due to women’s imbalanced postmenopausal physiology.

For males in Figure 14, the accuracy of the “bigger-age group” is higher than that of the “smaller-age group.” This difference is not related to hormones since men do not exhibit obvious menopause. It is more likely due to the data imbalance, as demonstrated by the imbalance factors of the “smaller-age group” at (1.54, 1.56, 1.57, 1.58, 1.59) and “bigger-age group” at (1.78, 1.70, 1.71, 1.67, 1.64). Note that a higher imbalance factor usually leads to higher accuracy.

In Table 6 for NVP, the best classifiers are Stack-LSTM (using feature set 2) for males and LSTM (using feature set 1) for females. This indicates that there is no single model and no single feature set that are best for both males and females.

It should be noted that by using Attention-LSTM with feature set 2, the accuracy only dropped by 0.96% for female FLD prediction and by 0.44% for male FLD prediction. The advantages in using feature set 2 include better efficiency in training/evaluation and more flexible prediction at any future time. Thus, if efficiency and flexibility are major concerns, we can sacrifice accuracy to a certain degree to achieve high efficiency and flexibility.

### Conclusions and Future Work

This study explored the use of a large health checkup dataset for FLD prediction in terms of current-visit and next-visit predictions. We used OPR and SFS for feature selection in CVP and then compared the results against expert-selected features. In our experiment with CVP, OPR was more efficient and provided comparable results with those obtained using SFS in terms of classification accuracy and the similarity between the automatically selected features and the expert-selected features.

For NVP, we propose two feature sets (feature sets 1 and 2) for various LSTM models. For females, the best accuracy of 81.90% was obtained when using feature set 1 for LSTM. For males, the best accuracy of 79.32% was obtained when using feature set 2 for LSTM. This indicates that the best models and best features are gender-dependent. However, it should be noted that feature set 2 is a much more compact representation; thus, it requires less time for training/evaluation, and there is no need for prior feature interpolation. Moreover, the model trained by feature set 2 is more flexible and it allows for FLD prediction at any time in the future.

In practice, NVP is much more valuable from the perspective of preventive medicine since whenever a positive prediction occurs, the physician can suggest lifestyle changes to prevent FLD at the next visit. To our knowledge, this is the first use of machine learning for NVP using a large-scale dataset.

Our immediate future work will focus on extending our LSTM-based NVP system to develop a comprehensive recommendation system, in which precise and personal recommendations will be given to prevent the potential future development of FLD, such as reduction in alcohol consumption, weight loss, and increased exercise. Such precise, personalized recommendations can be made based on patient clustering according to influential features. In general, such a system for preventive treatment can also be extended to other chronic or metabolic syndrome diseases, as long as we have a large dataset that covers many years for longitudinal studies.

## Appendix

Multimedia Appendix 1 Parameters of training options for the several variants of long short-term memory (LSTM).

## Abbreviations

AFLD: alcohol-related fatty liver disease
AUROC: area under the receiver operating characteristic curve
biLSTM: bidirectional long short-term memory
CART: classification and regression trees
CVP: current-visit prediction
FLD: fatty liver disease
GNB: Gaussian naive Bayes
IoU: intersection over union
KNNC: k-nearest neighbor classification
LOO: leave one out
LR: logistic regression
LSTM: long short-term memory
NAFLD: nonalcoholic fatty liver disease
NFLD: no fatty liver disease
NVP: next-visit prediction
OPR: one-pass ranking
RF: random forest
SFS: sequential forward selection
SVM: support vector machine
