# Supplementary material for: Current-Visit and Next-Visit Prediction for Fatty Liver Disease With a Large-Scale Dataset: Model Development and Performance Comparison
Source: JMIR Med Inform. 2021 Aug 12;9(8):e26398. doi: 10.2196/26398 (PMC8391752; doi:10.2196/26398)
Supplement: Multimedia Appendix 1 [file medinform_v9i8e26398_app1.docx]

**Multimedia Appendix 1. Parameters of training options for the several variants of long short-term memory (LSTM).**

The parameters of the option were divided into two cases: classifier and training.

- The dataset:
  - 70% for training in which 10% for validation
  - 30% for test
- The layers:
  - - The input size of sequenceInputLayer is 24
    - The number of numHiddenUnits is 100
    - The value of ’OutputMode’ is ’last’
    - The number of output class is 2
- The trainingOptions:
  - - The solver (known as loss function) for training network is ’rmsprop’
    - The ’LearnRateSchedule’ is ’piecewise’
    - The ’LearnRateDropPeriod’ is 2
    - The ’InitialLearnRate’ is 0.001
    - The ’ExecutionEnvironment’ is ’auto’
    - The ’GradientThreshold’ is 1
    - The iterator for training is 100
    - The ’MiniBatchSize’ as
    - If classifier for OPR is 91
    - If model for training of feature set 1 is 267
    - If model for training of feature set 2 is 144
    - The ’MaxEpochs’ of setting as
    - If classifier for OPR is 1
    - If model for training, the value is set to 10
